# Crystal structure of endo-β-*N*-acetylglucosaminidase HSα

**DOI:** 10.1107/S2053230X26001214

**Published:** 2026-02-28

**Authors:** Ikuya Kurauchi, Kazuki Okura, Chie Hosokawa, Kazuo Ito, Ikuko Miyahara

**Affiliations:** a Department of Chemistry, Graduate School of Science, Osaka Metropolitan University, 3-3-138 Sugimoto, Sumiyoshi-ku, Osaka558-8585, Japan; b Department of Biology, Graduate School of Science, Osaka Metropolitan University, 3-3-138 Sugimoto, Sumiyoshi-ku, Osaka558-8585, Japan; Osaka University, Japan

**Keywords:** endo-β-*N*-acetylglucosaminidases, GH85, complex *N*-glycans

## Abstract

The crystal structure of endo-β-*N*-acetylglucosaminidase HSα (Endo HSα) was determined at 1.8 Å resolution, revealing that the enzyme is composed of five distinct domains. Domains I to III adopt a fold that is conserved among GH85 enzymes, with catalytic residues Asn216, Glu218 and Tyr252 corresponding to conserved positions, while Tyr282 is newly implicated in catalysis based on the Endo HSα structure. A long loop unique to Endo HSα constricts the active site in domain I. Domain IV represents a novel structural element that is not observed in other GH85 enzymes. Its glycan-binding model and structural similarity to known sugar-binding domains play a role in substrate recognition. The minimal contacts with other domains allow it to remain flexible, accommodating bulky substrates at the active site. These features provide insights into the structural basis for substrate specificity and expand the structural diversity of the GH85 family.

## Abstract 2

Structural analysis of the GH85 enzyme endo-β-*N*-acetylglucosaminidase HSα reveals a novel domain that is potentially involved in complex *N*-glycan recognition.

## 1. Introduction

Endo-β-*N*-acetylglucosaminidase is an enzyme that hydrolyses the glycosidic bond between chitobiose units (GlcNAc β-1,4-GlcNAc) located in the core region of *N*-glycans on glycoproteins, thereby releasing the glycan chain. These enzymes are widely distributed across diverse organisms, including bacteria, fungi, plants and animals. Based on amino-acid sequence similarity, these enzymes are classified into glycoside hydrolase families GH18 and GH85. Endo-β-*N*-acetylglucosaminidase (Endo) H, originally isolated from *Streptomyces plicatus* (Tarentino & Maley, 1974 ▸; Tarentino *et al.*, 1974 ▸), belongs to the GH18 family and is a standard tool for the removal of high-mannose/hybrid glycans from glycoproteins. In contrast, Endo H does not act on the complex *N*-glycans typically found in mammalian glycoproteins (Maley *et al.*, 1989 ▸. The commercially available enzyme Endo M, originally isolated from *Mucor hiemalis*, also belongs to the GH85 family. Despite its pronounced activity towards high-mannose substrates, it retains substantial activity towards biantennary complex *N*-glycans (Yamamoto *et al.*, 1994 ▸). In addition to its hydrolytic activity, Endo M catalyzes transglycosylation, a property that has been applied to the synthesis of various glycopeptides (Yamamoto *et al.*, 1997 ▸; Haneda *et al.*, 1998 ▸). However, it does not act on *N*-glycans bearing core fucose or those with three or more antennae, and efficient glycan release requires prior digestion of the glycoprotein. In contrast, Endo HS, which is found in human saliva (Ito *et al.*, 1992 ▸; Ito, 2006 ▸), does not act on high-mannose and hybrid *N*-glycans; it specifically acts on complex *N*-glycans containing core fucose and three or more antennae, and it can release glycan chains from glycoproteins without denaturation (Ito *et al.*, 1993 ▸). As a member of the GH85 family, Endo HS also exhibits transglycosylation activity (Ito *et al.*, 2006 ▸; Ito, 2006 ▸,). Fairbanks (2017 ▸) provided a comprehensive summary of GH18 and GH85 endo-β-*N*-acetylglucosaminidases such as Endo H and Endo M, and described the structural and catalytic features of the enzymes in detail, including their catalytic residues and proposed reaction mechanisms. Although several studies based on the crystal structures of GH18 endo-β-*N*-acetylglucosaminidases have been reported in recent years (Trastoy *et al.*, 2021 ▸; Hsieh *et al.*, 2024 ▸; Sastre *et al.*, 2024 ▸), structural analyses of GH85 enzymes remain limited to Endo A and Endo D (Ling *et al.*, 2009 ▸; Yin *et al.*, 2009 ▸; Abbott *et al.*, 2009 ▸), both of which specifically act on high-mannose *N*-glycans. Endo HS was genetically cloned, and a purified form of Endo HSα2 lacking its N-terminal transmembrane domain exhibited enzymatic activity towards complex *N*-glycans similar to that of native Endo HS (Ito, 2014 ▸). The amino-acid sequence of Endo HSα2 contains a conserved GH85 domain, followed by a C-terminal extension of more than 250 residues that may be responsible for its unique enzymatic features, although its three-dimensional structure remains unresolved. This study presents the crystal structure of Endo HSα2 and aims to elucidate how the unique domains of Endo HSα2 contribute to its distinct enzymatic properties by comparing its structure with those of Endo A and Endo D.

## 2. Materials and methods

### 2.1. Protein expression and purification

The expression and purification of Endo HSα2 with a deleted N-terminal transmembrane region (hereafter referred to as Endo HSα) were performed as described by Ito (2014 ▸). Selenomethionine-labeled Endo HSα2 (SeMet Endo HSα) was produced using a selenomethionine-supplemented medium in accordance with established protocols (Doublié, 1997 ▸), and was subsequently purified in the same manner as Endo HSα.

### 2.2. Crystallization

The initial crystallization screening of Endo HSα was performed using a Mosquito liquid-handling robot (SPT Labtech). Plate-like crystals suitable for X-ray diffraction experiments were obtained within two weeks by the hanging-drop vapor-diffusion method under conditions consisting of 100 m*M* bis-Tris pH 5.5, 2.0 *M* ammonium sulfate. SeMet Endo HSα was also successfully crystallized under similar conditions. The crystals were cryoprotected in reservoir solution with an additional 25%(*v*/*v*) glycerol and then directly plunged into liquid nitrogen. Crystallization information is summarized in Table 1 ▸.

### 2.3. Data collection and processing

Diffraction data for Endo HSα were collected on the AR-NE3A beamline of the Photon Factory. SAD data for SeMet Endo HSα were collected at the selenium peak wavelength on the X06A beamline of the Swiss Light Source. Data were indexed, integrated and scaled using *XDS* (Kabsch, 2010 ▸). Data-collection parameters and statistics are summarized in Table 2 ▸.

### 2.4. Structure solution and refinement

The structure of SeMet Endo HSα was solved by SAD phasing using *AutoSol* in *Phenix* (Liebschner *et al.*, 2019 ▸). *AutoSol* was used to identify 34 selenium sites. The initial model of SeMet Endo HSα was built with *AutoBuild* in *Phenix*. Molecular replacement for native Endo HSα was performed using *MOLREP* (Vagin & Teplyakov, 2010 ▸) with the SeMet Endo HSα structure as the search model. Structure refinement was carried out using *phenix.refine* in *Phenix* and manual model rebuilding was performed with *Coot* (Emsley *et al.*, 2010 ▸). Refinement was initially performed with non­crystallographic symmetry (NCS) restraints applied between the two molecules in the asymmetric unit. The NCS restraints were subsequently removed in the final stages of refinement. Residues 30–1009 were modeled to represent the full-length sequence of Endo HSα2. Due to weak or ambiguous electron density, several side chains could not be confidently assigned. The loop region between residues 172 and 179 lacked interpretable density and was therefore omitted from the model in both molecules present in the asymmetric unit. The final model was validated using *MolProbity* (Chen *et al.*, 2010 ▸). Refinement statistics are summarized in Table 3 ▸. Structural figures were created using *PyMOL* (version 1.8; Schrödinger).

## 3. Results and discussion

### 3.1. Overall structure of Endo HSα

**Figure 1:**
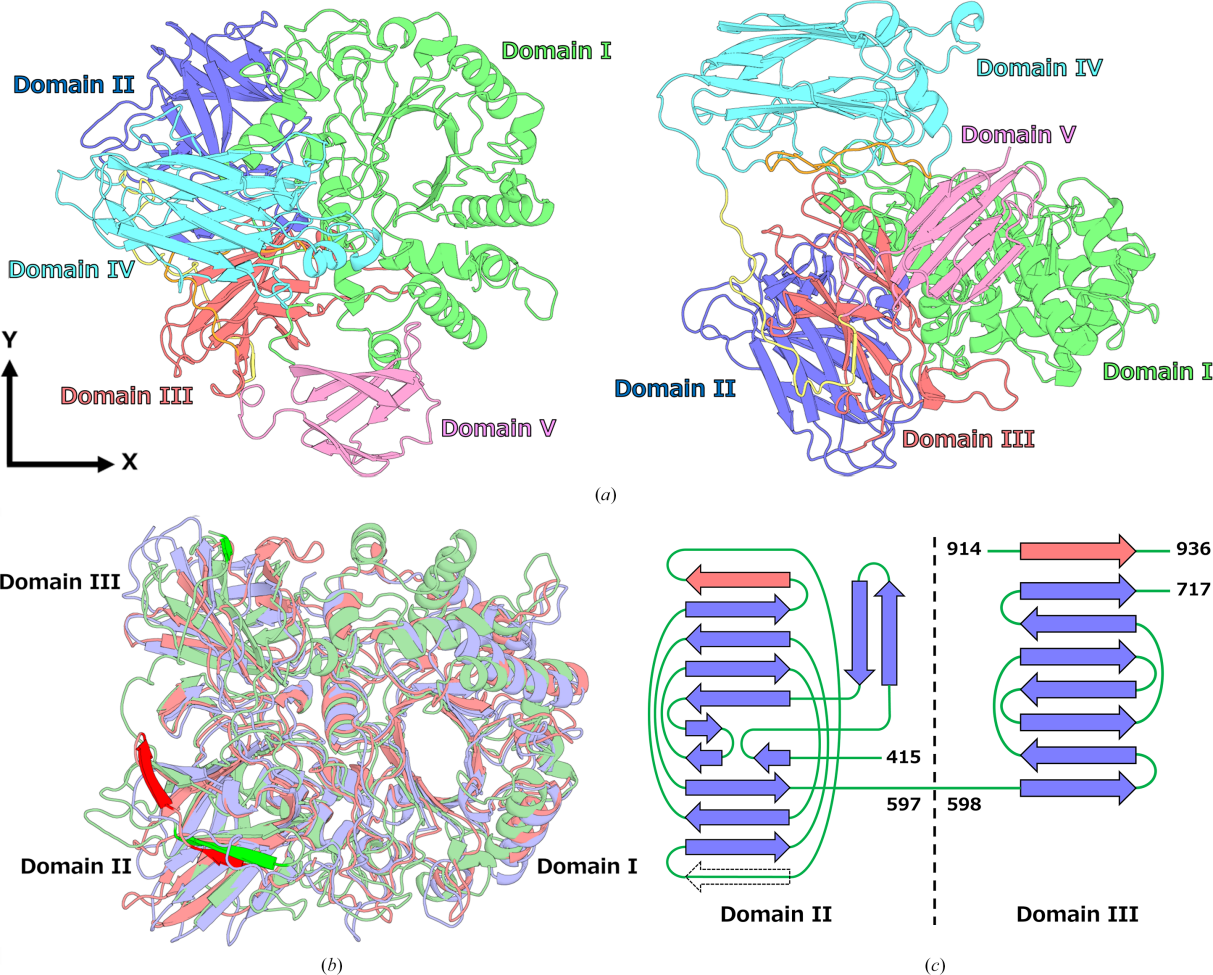

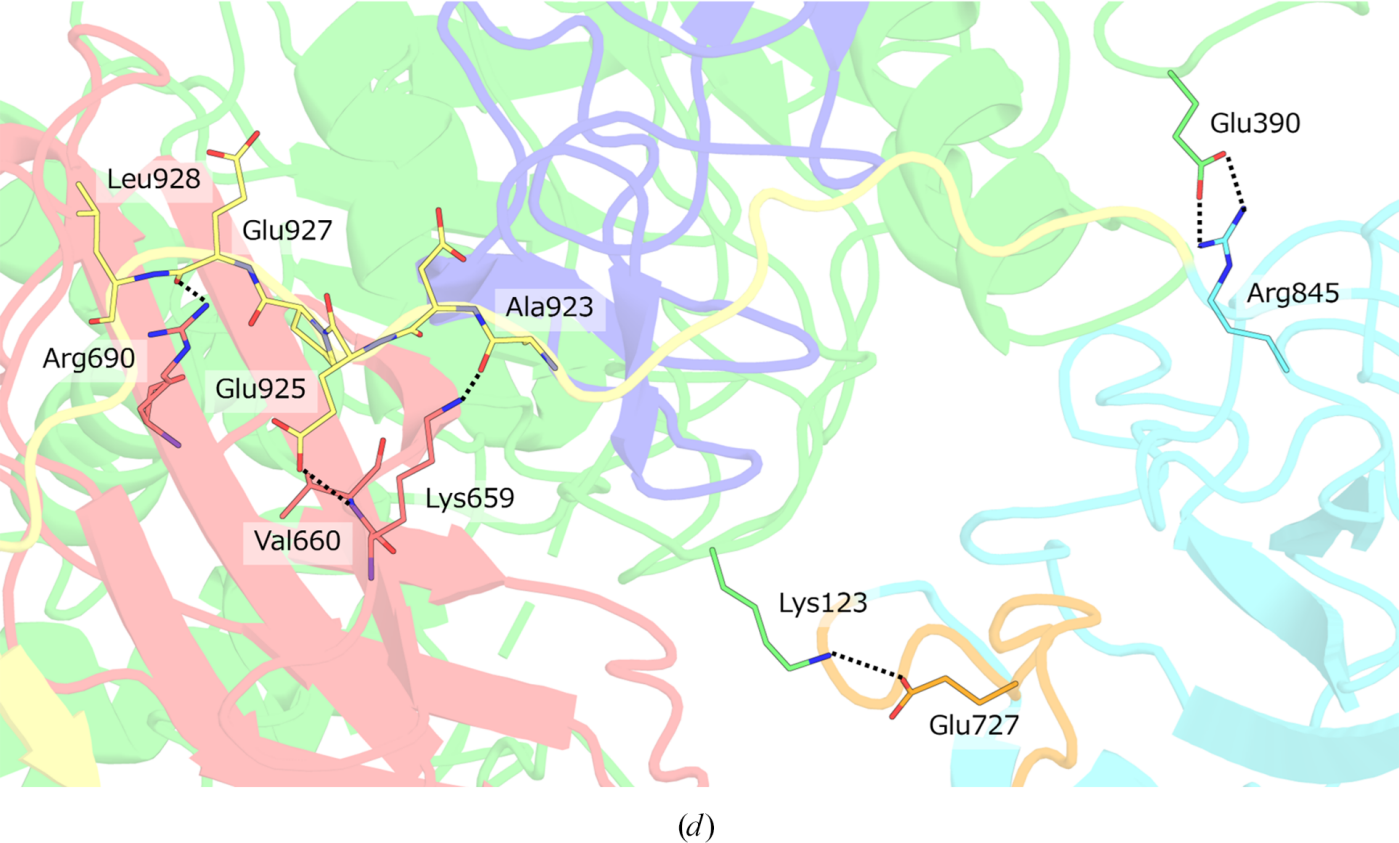
The overall structure of Endo HSα. (*a*) Overall structure of Endo HSα shown in two orientations. The left panel displays a top-down view along the axis of the TIM barrel, which corresponds to domain I. The right panel shows a side view obtained by rotating the structure 90° around the *x* axis. Individual domains are color-coded as follows: domain I, green; domain II, blue; domain III, red; domain IV, cyan; domain V, pink. Two long loops connecting domain IV are highlighted, with the N-terminal side shown in orange and the C-terminal side in yellow. (*b*) Structural superposition of Endo A (PDB entry 3fha, red) and Endo D (PDB entry 2w92, purple) onto Endo HSα (green), based on domains I to III. This view is rotated 180° around the *x* axis from the right panel of (*a*). The regions with different numbers of β-strands among the three structures are highlighted in a dark color to emphasize the structural variation. (*c*) Topology diagrams of domains II and III. In domain II the additional β-strand observed in Endo A is indicated with a black dotted arrow, whereas the β-strand conserved in Endo A and Endo HSα but absent in Endo D is indicated with a red arrow. In domain III of Endo HSα, the β-strand formed by the region between domains IV and V is shown with a red arrow. (*d*) Close-up view of the domain interface between domain IV and adjacent domains. The residues involved in the interdomain interactions are highlighted. Domain colors are consistent with those shown in (*a*).

The overall structure of Endo HSα was determined with two molecules present in the asymmetric unit. The contact area between adjacent molecules in the crystal was small, suggesting that Endo HSα exists as a monomer. The two molecules in the asymmetric unit were nearly identical, with a root-mean-square deviation (r.m.s.d.) of 0.334 Å between C^α^ atoms. Endo HSα comprises five domains (domains I to V; Fig. 1 ▸*a*). Domain I adopts an (α/β)_8_ TIM-barrel fold. Domain II consists of two antiparallel β-sheets, comprising four and five β-strands, forming a sandwich structure. In addition, two short β-strands form a separate antiparallel β-sheet. Domain III incorporates a Greek-key motif, and eight β-strands, including the β-strand formed in the region between domains IV and V, assemble into a β-barrel structure. As noted in Section 1, the structures of only two GH85 enzymes (Endo A and Endo D) have been determined. Although the amino-acid sequence identity of domains I to III in Endo HSα is relatively low (23% with Endo A and 20% with Endo D), structural superposition using Endo A (PDB entry 3fha) and Endo D (PDB entry 2w92) reveals a high degree of agreement in their tertiary structures, indicating a conserved core architecture within the GH85 family (Fig. 1 ▸*b*). However, Endo A contains an additional β-strand in domain II, whereas Endo D lacks one β-strand. Furthermore, domain III of Endo HSα includes a β-strand formed by the region between domains IV and V, resulting in a β-sheet with one additional β-strand compared with the corresponding domains in Endo A and Endo D (Fig. 1 ▸*c*).

Domain IV is a unique structure that is not found in other GH85 enzymes, comprising of nine β-strands forming two jelly-roll domains. It is located above the TIM barrel of domain I and is connected to domain III via an extended loop, resulting in limited contact with other domains. Notably, side-chain interactions are observed between Gly727 (domain IV) and Lys123 (domain I), and between Arg875 (domain IV) and Glu390 (domain III) (Fig. 1 ▸*d*). A *DALI* search, which compares protein structures based on distance-matrix alignment (Holm, 2022 ▸), revealed that domain IV shares structural similarity to the *O*-antigen polysaccharide-binding domain of the ABC transporter from *Aquifex aeolicus* (PDB entry 8dku; Spellmon *et al.*, 2022 ▸), with a *Z*-score of 7.8 and an r.m.s.d. of 3.3 Å, suggesting a potential role in sugar-chain recognition. However, the residues forming the sugar-binding site in PDB entry 8kdu are not conserved in domain IV, indicating that the similarity reflects a shared overall fold rather than a conserved binding mode.

Domain V adopts a Greek-key motif comprising seven β-strands arranged into two antiparallel β-sheets that form a barrel structure. A *DALI* search was carried out that identified structural similarity between domain V and the C-terminal domain of gingipain B from *Porphyromonas gingivalis* (PDB entry 5ag8; de Diego *et al.*, 2016 ▸), with a *Z*-score of 6.1 and an r.m.s.d. of 2.6 Å. Sequence analysis using the Pfam database (Paysan-Lafosse *et al.*, 2025 ▸) predicted that domain V corresponds to a C-terminal sorting domain of the secretion system. The enzymatic activity remains intact in mutants lacking this domain.

### 3.2. Active-site structure of Endo HSα

**Figure 2:**
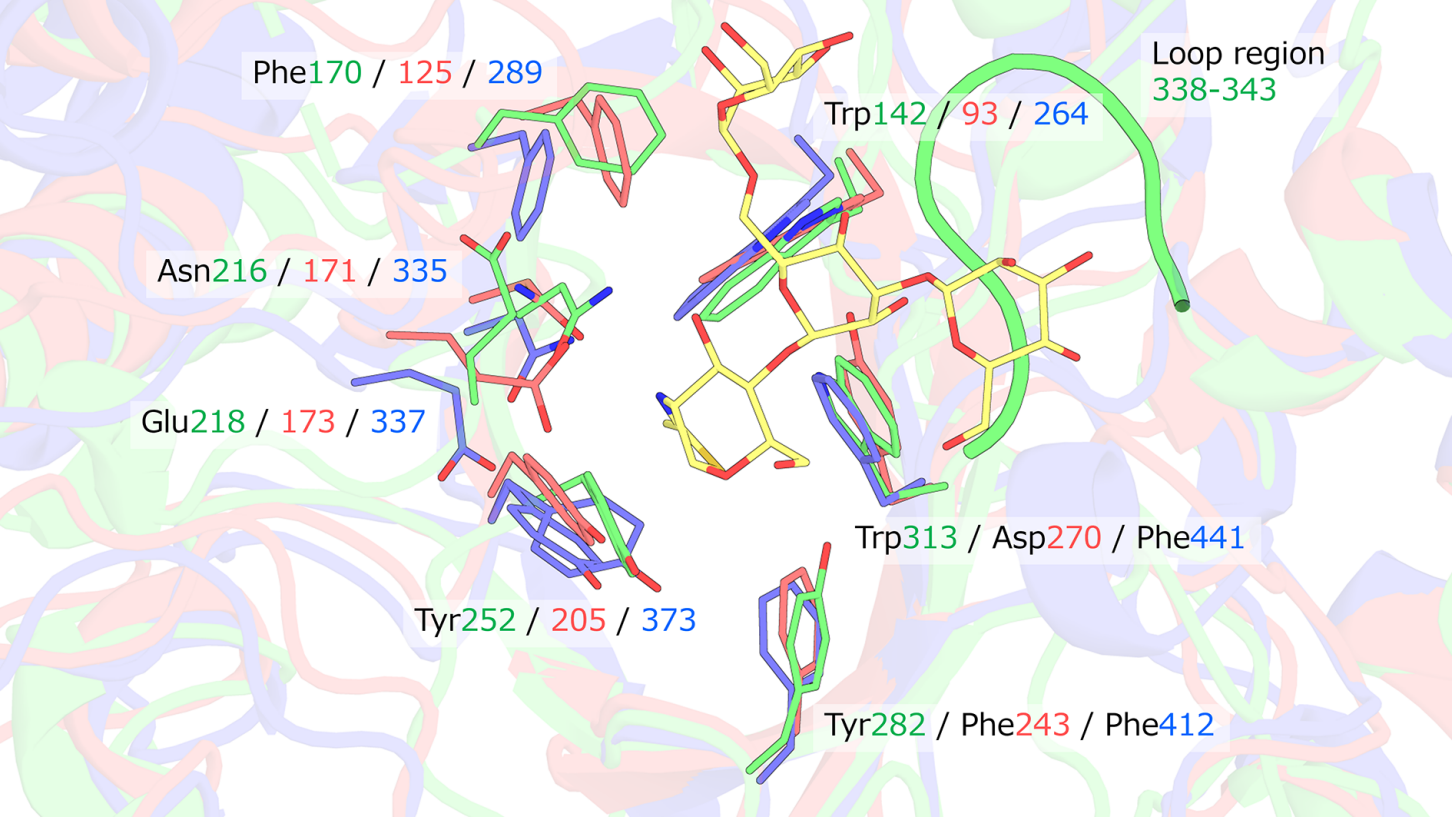
The active site is shown on the basis of the structural superposition of Endo HSα (green) and Endo D (PDB entry 2w91, blue) onto Endo A (PDB entry 3fhq, red) using domain I as the alignment reference. The catalytic residues and amino acids forming the hydrophobic pocket are depicted in stick representation, with residue numbers corresponding to each respective enzyme. The Man_3_GlcNAc-thiazoline bound to Endo A is shown as yellow sticks.

**Figure 3:**
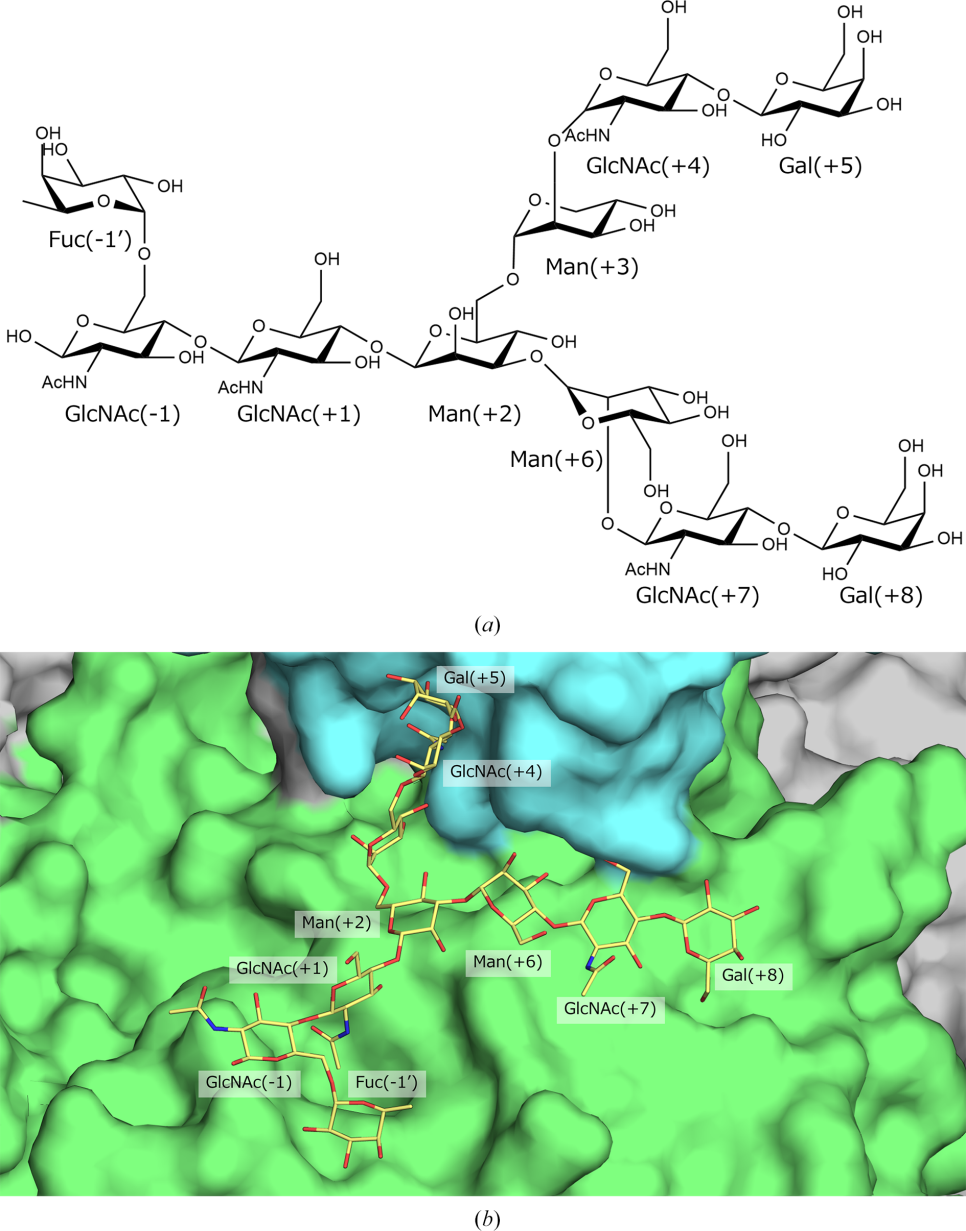
Results of docking simulation. (*a*) Structure of the biantennary complex *N*-glycan used for docking and (*b*) docked conformation of the N-linked glycan. Endo HSα is shown in surface representation, with domains I, II and IV represented in green, gray and cyan, respectively. The N-linked glycan is displayed in stick representation.

According to CAZy, a curated database that classifies carbohydrate-active enzymes based on sequence and structural features (Drula *et al.*, 2022 ▸), the Asn, Tyr and Glu residues are conserved among GH85 family enzymes and play critical roles in catalysis. Fig. 2 ▸ shows a superposition of domains I of Endo HSα and Endo D (PDB entry 2w91 chain *A*) onto domain I of the Man_3_GlcNAc-thiazoline-bound form of Endo A (PDB entry 3fhq chain *A*). The conserved positioning of the three catalytic residues implies that Endo HSα follows the same reaction mechanism as proposed for Endo A and Endo D, in which the substrate is hydrolyzed via the formation of an oxazoline intermediate (Yin *et al.*, 2009 ▸; Abbott *et al.*, 2009 ▸). In Endo A, the O^δ^ atom of Asn forms a hydrogen bond to the N2 atom of the oxazoline analog, whereas in Endo D the N^δ2^ atom of Asn interacts with the same site. Although the structure of Endo HSα lacks a bound substrate analog, the conserved catalytic configuration supports a similar mechanistic framework. However, it remains unclear whether the Asn residue adopts the same hydrogen-bonding mode as in Endo A or Endo D. In addition, the substrate-binding hydrophobic pocket is formed by residues with similar locations and properties across these enzymes. In contrast, several structural features distinguish Endo HSα from Endo A and Endo D. Endo HSα has Tyr282 near the catalytic Tyr252, whereas Endo A and Endo D have Phe at the equivalent position. To probe the role of Tyr282, the Tyr282Phe mutant was expressed and assayed as described by Ito (2014 ▸), and showed only minimal activity compared with Endo HSα (Supplementary Fig. S1), indicating that Tyr282 is essential for catalysis and/or substrate recognition. Furthermore, the loop region near the active site of Endo HSα is longer than the corresponding loops in Endo A and Endo D. This extended loop appears to narrow the space around the active site, potentially preventing the binding of bulky high-mannose glycans. Docking simulations with Endo HSα and a biantennary complex *N*-glycan (up to galactose; Fig. 3 ▸*a*) generated via *GLYCAM* (Kirschner *et al.*, 2008 ▸) were performed using *AutoDock Vina* (Eberhardt *et al.*, 2021 ▸). Endo HS is known to exhibit enzymatic activity towards complex *N*-glycans up to galactose (Ito *et al.*, 1993 ▸; Ito, 2006 ▸). We selected the model that exhibited high binding affinity and positioned the hydrolytic cleavage site near the catalytic residues (Fig. 3 ▸*b*). A distinct binding pocket for the core fucose residue was identified. In this model, one glycan antenna reached the boundary between domains I and domain IV, whereas the other extended into domain IV, suggesting a role for domain IV in substrate recognition. The glycan in this model includes only galactose residues; if sialic acid was present beyond this point, it would likely clash with domain IV. Considering that the presence or absence of sialic acid does not significantly affect the enzymatic activity of Endo HS (Ito *et al.*, 1993 ▸; Ito, 2006 ▸), a specific recognition site for sialic acid may not exist. Nevertheless, the enzyme remains active even in the presence of sialylated glycans, indicating that sufficient space must be available to accommodate sialic acid. As indicated in Section 3.1, domain IV makes only limited and weak contacts with other domains, suggesting that this domain may reposition to accommodate bulky substrates.

## 4. Conclusion

In this study, the crystal structure of Endo HSα was determined for the first time at 1.8 Å resolution, revealing the unique spatial arrangement of domain IV, which is not found in any of the PDB-deposited structures of other GH85 family enzymes. Structural similarity, as assessed by the *DALI* server, and sugar-chain binding models suggest that domain IV contributes to substrate recognition. The weak interaction of domain IV with other domains allows a flexible conformation that facilitates access to the active site by a bulky substrate. These distinctive features may underlie the ability of Endo HSα to act on native glycoproteins. In the future, atomic-level structural analysis of Endo HSα complexes with substrate analogs and glycoproteins will be essential to elucidate the detailed mechanism of substrate recognition.

## Supplementary Material

PDB reference: endo-β-*N*-acetyl­glucosaminidase HSα, 9x1i

Supplementary Fig. S1. DOI: 10.1107/S2053230X26001214/nw5136sup1.pdf

## Figures and Tables

**Table 1:** Crystallization

Method	Hanging-drop vapor diffusion
Temperature (K)	293
Protein concentration (mg ml^−1^)	10
Buffer composition of protein solution	10 m*M* sodium/potassium phosphate pH 6.5
Composition of reservoir solution	100 m*M* bis-Tris pH 5.5, 2.0 *M* ammonium sulfate
Volume and ratio of drop	4 µl, 1:1 ratio of protein:reservoir solution
Volume of reservoir (µl)	200

**Table 2:** Data collection and processing Values in parentheses are for the outer shell.

	Endo HSα	SeMet Endo HSα
Wavelength (Å)	1.000	0.9800
Temperature (K)	100	100
Rotation range per image (°)	0.25	0.5
Total rotation range (°)	180	360
Space group	*P*2_1_2_1_2_1_	*P*2_1_2_1_2_1_
*a*, *b*, *c* (Å)	71.93, 135.6, 238.9	71.7, 135.4, 237.1
α, β, γ (°)	90, 90, 90	90, 90, 90
Mosaicity (°)	0.22	0.11
Resolution range (Å)	49.7–1.81 (1.84–1.81)	49.5–1.95 (2.02–1.95)
Total No. of reflections	1402574 (63436)	2272258 (223609)
No. of unique reflections	212496 (10040)	325504 (32623)†
Completeness (%)	99.8 (96.4)	99.8 (99.9)
Multiplicity	6.6 (6.3)	7.0 (7.2)
〈*I*/σ(*I*)〉	12.9 (2.8)	10.9 (2.1)
*R*_r.i.m._	0.093 (0.748)	0.056 (0.405)
Overall *B* factor from Wilson plot (Å^2^)	21.2	23.7

† Values calculated without merging Friedel pairs.

**Table 3:** Structure solution and refinement Values in parentheses are for the outer shell.

Resolution range (Å)	39.05–1.81 (1.84–1.81)
Completeness (%)	99.80 (98.94)
No. of reflections, working set	212229 (20759)
No. of reflections, test set	10601 (6439)
Final *R*_work_	0.1959 (0.2664)
Final *R*_free_	0.2419 (0.3102)
No. of non-H atoms	
Protein	15077
Ion	12
Glycerol	30
Water	2378
Total	17464
R.m.s. deviations	
Bond lengths (Å)	0.007
Angles (°)	0.890
Average *B* factors (Å^2^)	
Protein	26.43
Ion	31.11
Glycerol	31.87
Water	34.25
Ramachandran plot	
Most favored (%)	96.95
Allowed (%)	2.94
Outliers (%)	0.01

## Data Availability

The atomic coordinates and structure factors have been deposited in the Protein Data Bank under accession code 9x1i.
