# Supplementary material for: Crystal structure of endo-β-N-acetylglucosaminidase HSα
Source: Acta Crystallogr F Struct Biol Commun. 2026 Feb 28;82(Pt 3):94–100. doi: 10.1107/S2053230X26001214 (PMC12961652; doi:10.1107/S2053230X26001214)
Supplement: Supplementary file 1 [file f-82-00094-sup1.pdf]

STRUCTURAL BIOLOGY COMMUNICATIONS

Volume 82 (2026)

Supporting information for article:

Crystal structure of endo-β-*N*-acetylglucosaminidase HSα

Ikuya Kurauchi, Kazuki Okura, Chie Hosokawa, Kazuo Ito and Ikuko Miyahara

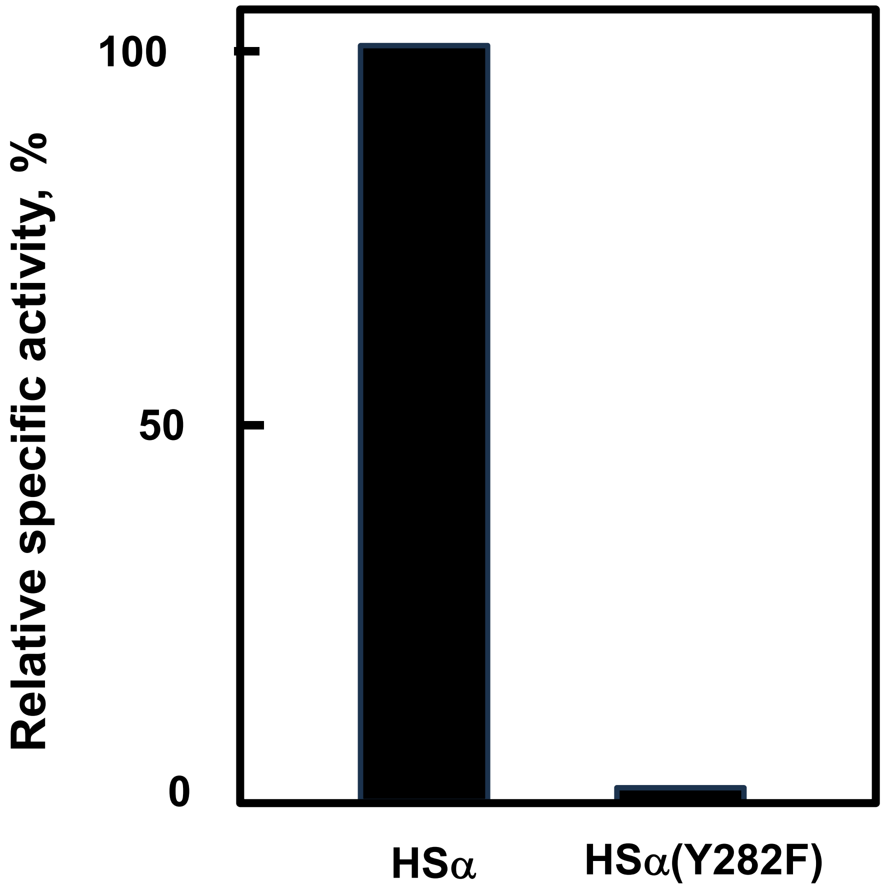

Figure S1 Relative specific activity of the Tyr282Phe mutant of Endo HSα. The Tyr282Phe mutant was generated by inverse PCR and expressed in *E.coli.* and protein expression was confirmed by SDS–PAGE. Deglycosylation activity was assayed as described previously (Ito, 2014) using human transferrin as a substrate. The relative specific activity of Endo HSα(Tyr282Phe) compared with Endo HSα is shown.
